# 3-D Modelling and Experimental Comparison of Reactive Flow in Carbonates under Radial Flow Conditions

**DOI:** 10.1038/s41598-017-18095-2

**Published:** 2017-12-18

**Authors:** Piyang Liu, Jun Yao, Gary Douglas Couples, Jingsheng Ma, Oleg Iliev

**Affiliations:** 1 School of Petroleum Engineering, China University of Petroleum (East China), QingDao, 266580 China; 2 Institute of Petroleum Engineering, Heriot-Watt University, Riccarton, Edinburgh EH14 4AS UK; 3 Fraunhofer Institute for Industrial Mathematics (ITWM), Kaiserslautern, 67663 Germany

## Abstract

We use a two-scale continuum model to simulate reactive flow and wormhole formation in carbonate rocks under 3-D radial flow conditions. More specifically, we present a new structure-property relationship based on the fractal geometry theory, to describe the evolution of local permeability, pore radius, and specific area with porosity variation. In the numerical calculation, to improve the convergence rate, the heterogeneous medium in question is extended by adding a thin layer of homogeneous porous medium to its inlet. We compare the simulation results with the available experimental observations and find that they are qualitatively consistent with each other. Additionally, sensitivity analysis of the dissolution process with respect to acid injection rate and rock heterogeneity, including heterogeneity magnitude and correlation length, is presented.

## Introduction

Reactive flow plays an important role in a variety of geological, scientific, and engineering processes. These include spontaneous processes such as karstification^1,2^, melt migration^3,4^, diagenesis^5^, sinkhole formation^6,7^, environmental contaminant transport^8–10^, as well as anthropogenic processes such as geologic sequestration of carbon dioxide^11–17^, disposal of nuclear wastes^18^, and injection of acid in petroleum reservoirs^19,20^.

In order to understand the dissolution process, numerous core experiments have been carried out using a variety of reactant-medium systems, for example, plaster dissolved by water^21,22^, under-saturated salt solution dissolving salt packs^23,24^, and carbonate rock treated with acid^25–27^. Moreover, the effects of various physical and chemical characteristics such as core geometry^28^, core dimension^29^, temperature^30^, reaction products^31^, and chemical kinetics^25^, on dissolution dynamics have also been investigated through core flood experiments. These experimental studies indicate that one of a characteristic set of dissolution patterns will form, depending on different injection rates of the reactant. For example, at very low injection rates, the reactant can be completely consumed before it penetrates deeply into the core. As a result, the dissolution is restricted in the region near the fluid entrance, and the face dissolution pattern is formed. At the other extreme, when the reactant is injected at very high rates, it invades nearly all parts of the pores of the rock, increasing the porosity uniformly, leading to the uniform dissolution pattern. Between these two extremes, as the injection rate increases, conical, wormhole and ramified dissolution patterns are formed. By measuring the volume of acid required to increase the core effective permeability by a certain factor, i.e. the breakthrough volume *PV*_*BT*_, these studies also observed that the least amount of acid is required when the wormhole dissolution pattern is formed. For carbonate reservoir stimulation, on which the goal is to increase the effective connectivity between the wellbore and the distant rock matrix, the creation of deep and thin wormholes, which needs a minimal volume of injected acid, is economically favorable^20,32^. Therefore, there is a practical motivation for understanding the sets of characteristics and conditions which lead to the development of wormholes.

Several models have been proposed over the last few decades to investigate the acidization process and wormhole formation. Maheshwari, *et al*.^33^ provide a good summary and classify the present models into four types: (1) dimensionless model^22,34,35^; (2) capillary tube model^36–38^; (3) network model^15,39–42^ and (4) continuum model^20,24,33,43–56^. Because the continuum model is advantageous at forecasting the dissolution patterns observed in experiments, it has been widely used and continuously extended over the last few years, to replicate the actual acidizing operation. These extensions include simulating the wormhole formation under the radial flow condition^44,57^, using various types of injected acids^50,51,53,58,59^, for various reservoir characteristics such as the presence of fractures^20,55^ and vugs^46^, different completion methods^47^, and various reservoir temperatures^45,48^.

Although all these studies bring important insights about the dissolution process, there are some issues that still remain to be further investigated. Firstly, the actual acidizing treatments are conducted by injecting acid into the formation through a wellbore. In this case, the flow is radial and the nominal velocity decreases rapidly as acid flows away from the wellbore. Therefore, 3-D radial models are needed to simulate the wormhole formation at exact downhole environments. However, in the literature, very few studies^57,60^ have been performed under a 3-D radial flow condition to understand the dissolution process.

Secondly, as dissolution progresses, some minerals are dissolved by the acid, which results in a local porosity increase. It is difficult to correlate the changes in local permeability, pore radius, and specific surface area to porosity changes. One possible way is using the pore-scale model based on detailed textural analysis of the real rock material. However, it is computationally unfavourable to use this approach as the scale of a wormhole is much larger than the scale of pores. Alternatively, some empirical equations, for example the Carman-Kozeny equation, can be used to relate the rock properties with structure. As mentioned by Maheshwari and Balakotaiah^49^, the choice of the structure-property relations affects the simulation of the dissolution process significantly. In most of the published studies^43,44,54,57,61^, a modified Carman-Kozeny equation was used, in which the exponential parameter in the Carman-Kozeny equation is modified from a constant value of 2 to a variable *β*. However, parameter values involved in these relations are specific to a rock-acid system and should be determined previously through core acidizing experiment^49^. To improve the accuracy of the single parameter correlation, Maheshwari and Balakotaiah^49^ proposed a two-parameter structure-property relation by adding an exponential variable to the ratio of changed porosity to initial porosity *ϕ*/*ϕ*_0_. But, this extension has no physical meaning and it is difficult to measure the coefficients in the laboratory^62^. In addition, numerous theoretical models and experimental observations indicate that the coefficients involved in these structure-property relations are not a constant value, but related to the porosity, microstructures of pores, and capillaries^63–65^. Therefore, a more reliable correlation is required to describe the dissolution process accurately.

To address the above mentioned issues, this work adopts the two-scale continuum model developed by Panga, *et al*.^43^ which is extended to the 3-D radial flow condition by using a new analytic structure-property relation. The simulation results are compared with the available experimental observations as a validation. Additionally, a sensitivity analysis of the dissolution process with respect to acid injection rate and rock heterogeneity, including heterogeneity magnitude and correlation length, is presented.

## Results and Discussions

We first present the simulation result of the dissolution structure and compare it with the available experimental results obtained by Walle and Papamichos^66^. For this purpose, a cylindrical 3-D domain with a circular hole in the centre is considered. In Walle and Papamichos’ experiment, 15% HCl was injected radially into the Mons chalk core through the central borehole at a constant injection rate of 25 mL/min, which is the optimum injection rate leading to wormhole formation. The core is 20 cm in height, 20 cm in outer diameter, with a 2 cm inner hole diameter. The upper and lower surfaces of the core are sealed, and the injected acid is allowed to flow out through the circumferential boundary, where the backpressure is fixed. The flooding continues until acid breaks through the core, which is identified when the borehole pressure drops rapidly to the value of the backpressure. The parameter values used in the simulation shown here are the same as those in the experimental study. Since not all parameters used in the calculation are available in the experimental data, the values of unspecified or unknown variables, such as the average pore radius, and the specific surface area et.al., are taken as reported in a previous similar acidizing numerical simulation study^67^, and all of these numerical values can be found in Supplementary Table S1.

**Figure 1:**
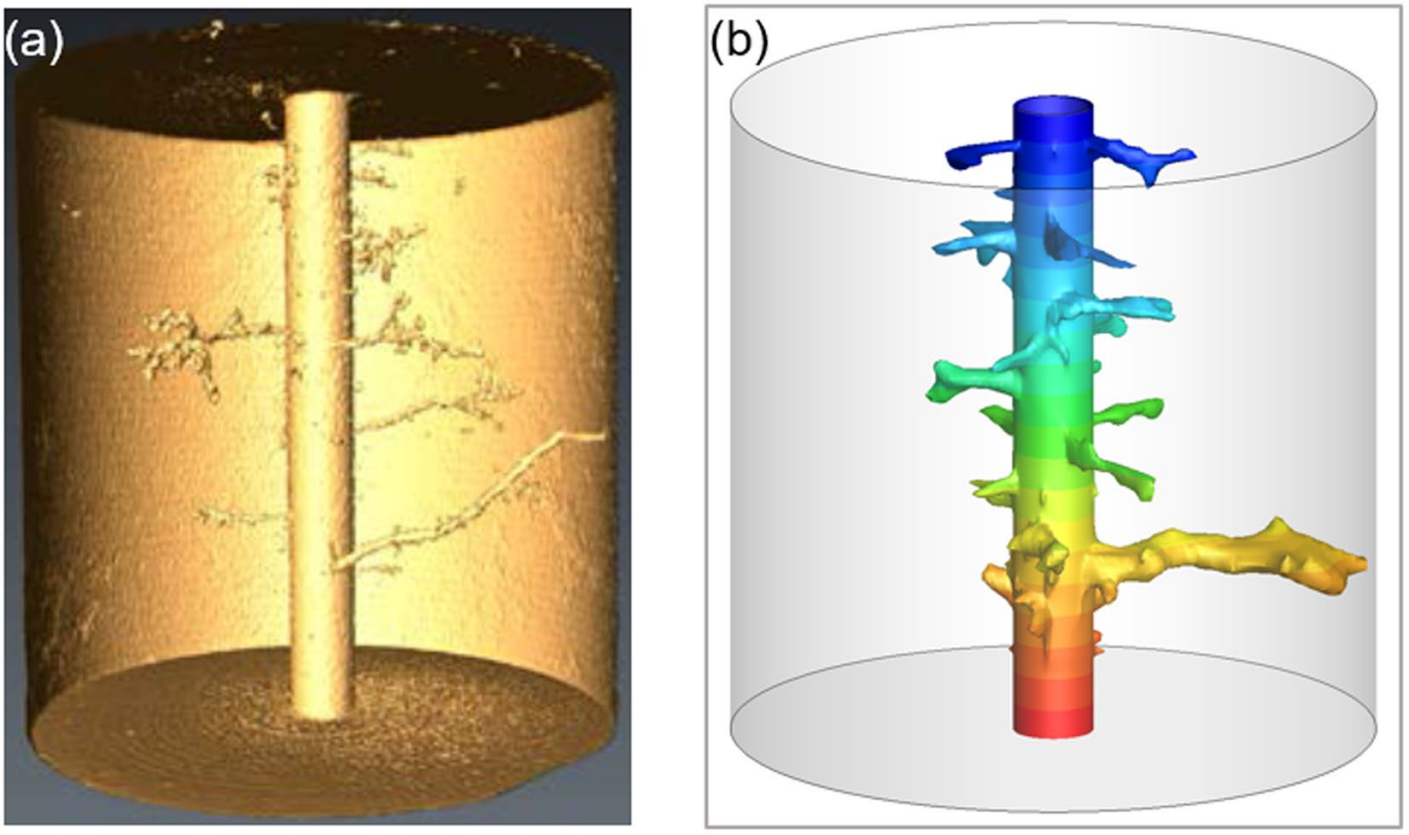
Comparison of wormhole structure obtained from (**a**) experiment by Walle and Papamichos^66^ and (**b**) simulation using the present method.

The comparison between the dissolution patterns obtained from the acid flooding experiment, and our simulation, is depicted in Fig. 1. It can be clearly seen that the simulation result is in good agreement with the experimental result in terms of the spatial characteristics. The slight deviation could be because of the differences in heterogeneity of the porosity between the rock used for experiments and the porous medium created for the numerical simulations.

Following validation of the model, we present a sensitivity analysis with respect to the injection rate, the heterogeneity magnitude of the rock, and the correlation length of the initial porosity field. In actual reservoirs, the thickness of the formation is usually less than the radius of the domain considered. Therefore, in the following analysis, a cylindrical core with external diameter of 5 cm, internal diameter of 0.5 cm, and height of 2 cm, which has similar aspect ratios as the actual reservoir, is used to perform the simulations. Other parameter values used in these simulations are the same as listed in Supplementary Table S1. All these values are fixed unless otherwise stated.

### Effect of acid injection rate

**Figure 2:**
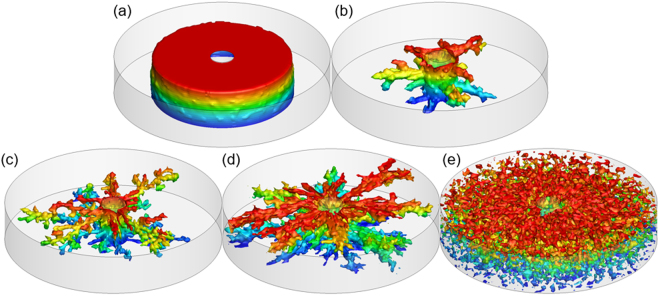
Effect of injection rate on dissolution patterns. (**a**) face dissolution at *v* = 0.0001 cm/s; (**b**) conical wormhole at *v* = 0.003 cm/s; (**c**) wormhole at *v* = 0.06 cm/s; (**d**) ramified wormhole at *v* = 3 cm/s; (**e**) uniform dissolution at *v* = 60 cm/s.

In the past few decades, various experimental and numerical studies have been performed to analyze the effect of injection rate on the dissolution process. For example, Fredd and Fogler^68^ investigated the effect of injection rate on the dissolution structure by conducting acidizing experiments on carbonate cores with HCl, and five types of typical dissolution patterns, named face dissolution, conical wormhole, dominate wormhole, ramified dissolution and uniform dissolution, are observed. Panga, *et al*.^43^, Kalia and Balakotaiah^44^, and Maheshwari, *et al*.^33^ obtained the same observations by numerical simulation under 2-D linear, 2-D radial, and 3-D linear flow conditions, respectively. As depicted in Fig. 2, these five types of dissolution patterns are also observed in our simulations by injecting the acid into 3-D cylindrical cores radially at different injection rates. In order to visualize the dissolution structure in the 3-D domain, the final porosity fields with porosity greater than 0.35 are shown in Fig. 2, as the maximum value of the initial porosity used in this simulation is 0.35. It should be noted that these pictures only show the approximate dissolution structure, because the areas where the initial porosity is low and has been dissolved, but does not have an altered porosity greater than 0.35, have not been displayed. (Note: the color in all dissolution patterns is only used to show the elevation).

When the acid injection rate is very low, the mass transfer rate is much smaller than the reaction rate. As a result, acid is consumed instantaneously at the fluid-solid interface and this leads to the face dissolution (Fig. 2(a)). With an increase of the injection rate, acid transport begins to be governed by both the advection and dispersion mechanisms. If dispersion still plays an important part in acid transport, the dissolution front will propagate in both radial and transverse directions leading to the formation of conical wormholes (Fig. 2(b)). When the magnitude of radial advection, transverse dispersion and reaction rate reaches such a status that the velocity of solute transporting to the dissolution front by advection and dispersion is exactly equal to the rate of acid consumption, acid preferentially flows into bigger pores and hence only these bigger pores keep growing with time, which results in some conducting narrow channels named dominate wormholes (Fig. 2(c)). As the acid injection rate further increases, the concomitant increase in advection velocity causes the total acid transport rate to be larger than the reaction rate, and hence the injected acid cannot be completely consumed at the dissolution front. In this case, the residual acid will be transported in all directions by the dispersion mechanism, which results in highly branched channels known as ramified wormholes (Fig. 2(d)). In the final extreme case of very high acid injection rate, the acid transports so fast that it has insufficient time to significantly react with the rock. As a result, acid reaches nearly all parts of the rock and increases the porosity throughout the rock approximately uniformly, leading to a uniform dissolution (Fig. 2(e)).

### Effect of heterogeneity magnitude

It is difficult to study the effect of rock heterogeneity on the dissolution process through experimentation. This is because the rock properties depend on the historical evolution of the specific rock sample over a period of time, and it is not possible to control natural rock samples to the degree of similarity required to address the underlying question. Conversely, through numerical simulation, we can easily keep other variables fixed and vary the heterogeneity magnitude to investigate its effect on the dissolution process. Several investigators have attempted to do this. For example, Kalia and Balakotaiah^67^ performed some 2-D simulations on porous media with different heterogeneities and observed that a medium degree of heterogeneity influences both the breakthrough volume and dissolution structure. Maheshwari, *et al*.^33^ studied the effect of heterogeneity magnitude on 3-D linear dissolution and found that a wormhole becomes highly branched and fractal in nature with heterogeneity magnitude increase. However, the effect of heterogeneity magnitude on 3-D radial dissolution, which is closer to an actual acidizing treatment in a reservoir, still remained to be investigated.

**Figure 3:**
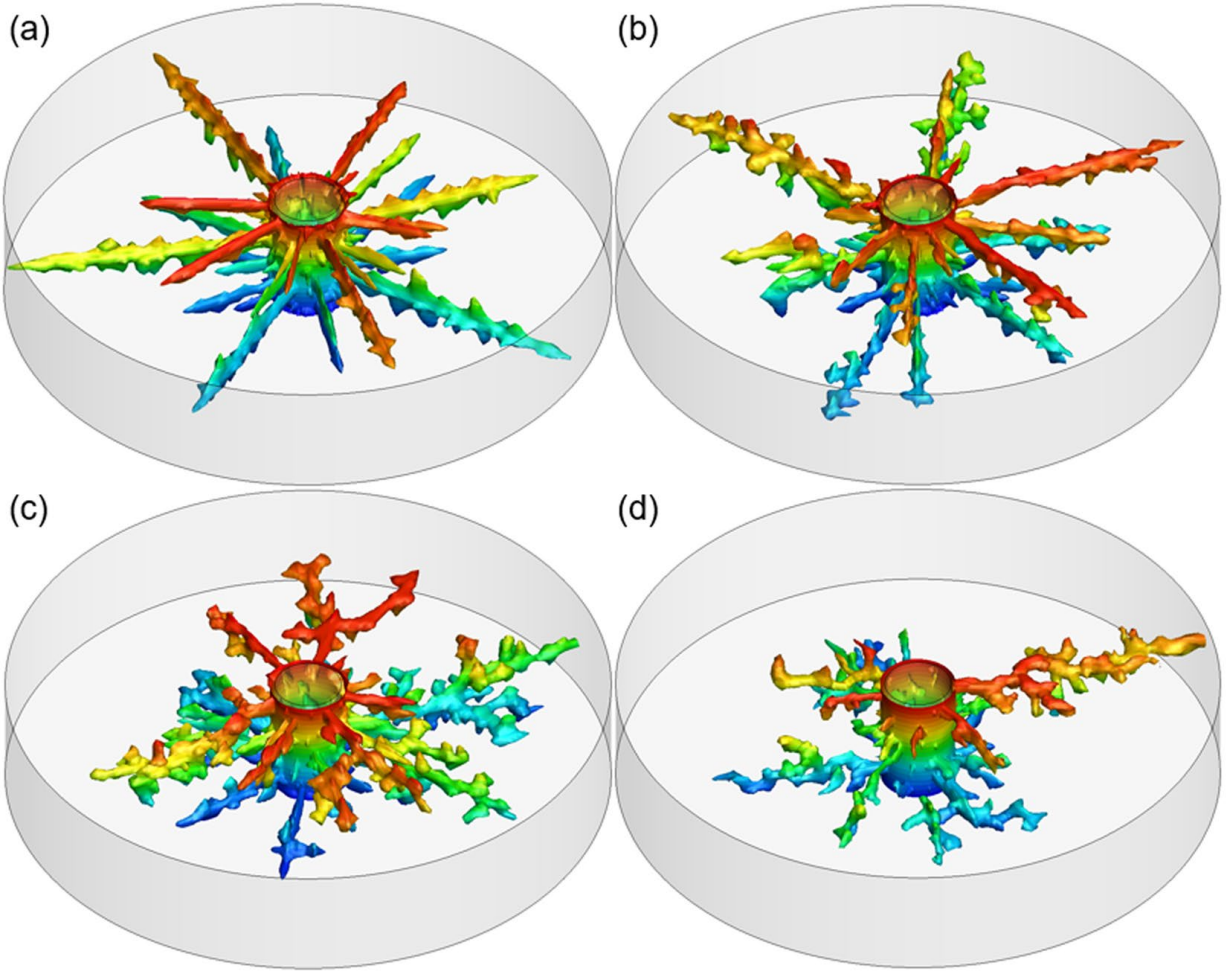
Effect of heterogeneity magnitude on wormhole structures. The correlation length *l* = 0.2 cm, and the heterogeneity magnitude are (**a**) ${\rm{\Delta }}\varphi =0.02$, (**b**) ${\rm{\Delta }}\varphi =0.05$, (**c**) ${\rm{\Delta }}\varphi =0.1$, (**d**) ${\rm{\Delta }}\varphi =0.15$.

The dissolution structures obtained from numerical simulation on 3-D cylindrical rocks with various heterogeneity magnitudes, at the optimum acid injection rate, are shown in Fig. 3. It can be seen that when the heterogeneity magnitude is low, more than one straight and smooth wormhole is formed by the time that acid breakthrough occurs (Fig. 3(a)). This is because in this case the permeability throughout the core is almost uniform. Therefore, the injected acid does not change its flow direction, and wormholes propagate at the same rate. It is easy to imagine that when no heterogeneity is present in the rock, the dissolution front will propagate stably and face dissolution will be observed. With an increase of the heterogeneity magnitude, wormholes become highly branched and the number of dominate wormholes decreases. At appropriate heterogeneity magnitude (Δ*ϕ* = 0.15 in this case, as shown in Fig. 3(c)), only one dominate wormhole forms and the growth of others is stopped because the one providing the least resistance to flow captures most of the acid.

### Effect of correlation length

Correlation length describes the spatial correlations between the pore spaces in porous media, which is defined here as the average distance between any two sites belonging to the same cluster of the pore space^69^. The importance of correlation length in petrophysical properties and fluid flow has been illustrated in many studies. Kalia and Balakotaiah^67^ defined an analogous parameter, named length scale, in investigating the effect of rock heterogeneity on the dissolution process. In their work, one porosity value is assigned to several interconnected grid cells, and the number of interconnected grids with the same porosity values is called the length scale. After introducing the length scale, the porosity field is divided into some uniform clusters, however, it is significantly different from the actual porosity distribution of the rock. Here, we investigate the effect of correlation length on dissolution process based on our new porosity generation method.

**Figure 4:**
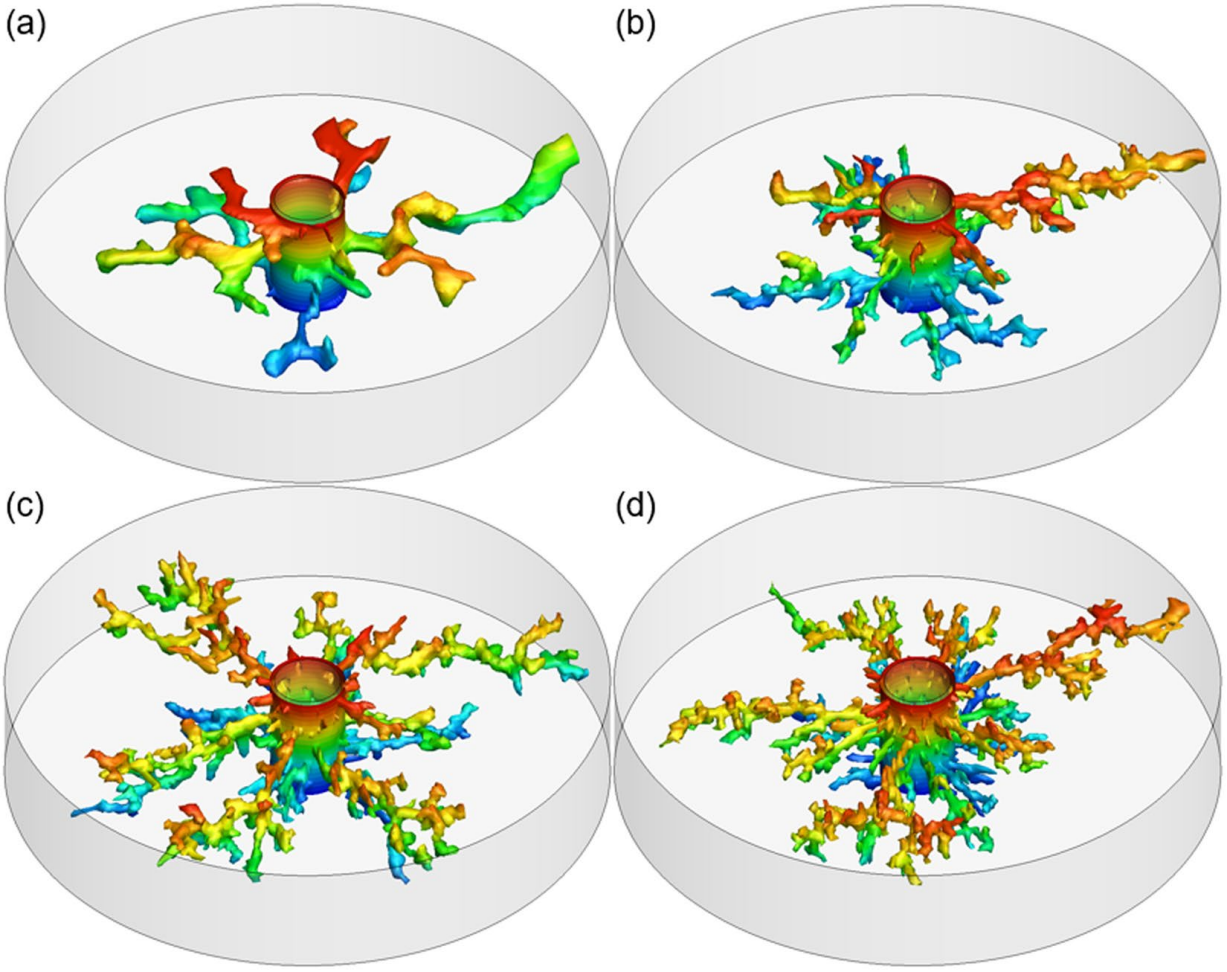
Effect of correlation length on wormhole structures. The heterogeneity magnitude ${\rm{\Delta }}\varphi =0.15$, and the correlation length are (**a**) *l* = 0.45 cm, (**b**) *l* = 0.2 cm, (**c**) *l* = 0.15 cm, (**d**) *l* = 0.1 cm.

Four porosity fields with different correlation length values of *l* = 0.45 cm, *l* = 0.2 cm, *l* = 0.15 cm, and *l* = 0.1 cm, are generated, respectively. The acid is injected into the core at the optimum injection rate. Figure 4 shows the dissolution structures from numerical simulations using these porosity fields. It can be seen that wormholes become highly branched with the decrease of correlation length, similar with the effect of heterogeneity magnitude. Another observation is that the wormhole tip diameter decreases with the decrease of correlation length. A similar effect, relative to wormhole diameter, resulting from varying the length scale, has been found by Maheshwari, *et al*.^33^. Additionally, the correlation length has almost no effect on the number of dominate wormholes. For all four cases in our study, only one dominate wormhole is formed. However, this conclusion may be broken if the correlation length is too high such that the rock is effectively homogeneous; in that case, face dissolution occurs.

## Methods

The two-scale continuum model presented by Panga, *et al*.^43^ is extended to simulate the reactive dissolution under a 3-D radial flow condition. It consists of a Darcy-scale model and a pore-scale model. The Darcy-scale model that describes the reactive transport of acid and the evolution of the rock can be expressed in the cylindrical coordinate system as follows:

1 $$(u,v,w)=-\frac{K}{\mu }(\frac{\partial P}{\partial r},\frac{1}{r}\frac{\partial P}{\partial \theta },\frac{\partial P}{\partial z})$$

2 $$\frac{\partial \varphi }{\partial t}+\frac{1}{r}\frac{\partial }{\partial r}(ru)+\frac{1}{r}\frac{\partial v}{\partial \theta }+\frac{\partial w}{\partial z}=0$$

3 $$\begin{array}{cc} & \frac{\partial }{\partial t}(\varphi {C}_{f})+\frac{1}{r}\frac{\partial }{\partial r}(ru{C}_{f})+\frac{1}{r}\frac{\partial }{\partial \theta }(v{C}_{f})+\frac{\partial }{\partial z}(w{C}_{f})\\ = & \frac{1}{r}\frac{\partial }{\partial r}(r\varphi {D}_{r}\frac{\partial {C}_{f}}{\partial r})+\frac{1}{r}\frac{\partial }{\partial \theta }(\frac{\varphi {D}_{\theta }}{r}\frac{\partial {C}_{f}}{\partial \theta })+\frac{\partial }{\partial z}(\varphi {D}_{z}\frac{\partial {C}_{f}}{\partial z})-\frac{{R}_{s}{R}_{c}{a}_{v}}{{R}_{s}+{R}_{c}}{C}_{f}\end{array}$$

4 $$\frac{\partial \varphi }{\partial t}=\frac{{R}_{s}{R}_{c}{a}_{v}{\alpha }_{c}}{({R}_{s}+{R}_{c}){\rho }_{s}}{C}_{f}$$

In order to be consistent with the experimental conditions, the initial and boundary conditions used to solve the above system of PDEs are considered as follows:

5 $${C}_{f}={C}_{0},\,{\rm{at}}\,t=0$$

6 $$-\frac{K}{\mu }\frac{\partial P}{\partial r}={u}_{0},\,\frac{1}{r}\frac{\partial P}{\partial \theta }=0,\,\frac{\partial P}{\partial z}=0,\,{\rm{at}}\,r={r}_{w}$$

7 $$u{C}_{f}-\varphi {D}_{r}\frac{\partial {C}_{f}}{\partial r}={u}_{0}{C}_{0},\,{\rm{at}}\,r={r}_{w}$$

8 $$P={P}_{e},\,\frac{\partial {C}_{f}}{\partial r}=0,\,{\rm{at}}\,r={r}_{e}$$

9 $$\frac{\partial P}{\partial z}=0,\,\frac{\partial {C}_{f}}{\partial z}=0,\,{\rm{at}}\,z=0\,{\rm{and}}\,z=h$$

10 $$P(r,0,z)=P(r,2\pi ,z),{C}_{f}(r,0,z)={C}_{f}(r,2\pi ,z)$$

In equations (1–10), *r*, *θ* and *z* are coordinate parameters; *u*, *v*, *w* are the components of Darcy velocity in *r*, *θ*, and *z* directions, respectively; *t* is the time; K is the permeability, which is calculated from a pore-scale model or determined by lab measurement; *μ* is the viscosity of the fluid phase; *P* is the pressure of the pore-fluid; *ϕ* is the porosity of the rock; *C*_*f*_ is the cup-mixing concentration of the solute in the fluid phase; *D*_*r*_, *D*_*θ*_ and *D*_*z*_ are the components of the effective diffusion vector in *r θ*, and *z* directions, respectively; *a*_*v*_ is the interfacial area available for reaction per unit volume of the medium; *R*_*c*_ is the mass transfer coefficient; *R*_*s*_ is the dissolution rate constant, with units of velocity; *α* is the dissolving power of the acid, defined as grams of dissolved solid per mole of acid reacted; and *ρ*_*s*_ is the density of the solid; *u*_0_ is the constant injection velocity; *C*_0_ is the acid concentration; *r*_*w*_ and *r*_*e*_ are the radius of the inlet and outlet boundaries, respectively. It should be noted that the model described above is for a first order irreversible reaction (valid for the case of HCl-CaCO_3_ reaction), but the model proposed is completely general and can be extended to other kinetics.

For a homogeneous system, the dissolution front propagates stably, leading to the face dissolution. In order to simulate the different dissolution patterns, the heterogeneity needs to be introduced into at least one of the porosity field, permeability field and injection scheme. Since real carbonate rocks are spatially heterogeneous, the most commonly used method in numerical simulation is to adopt a heterogeneous porosity field, which is usually generated by perturbing the initial mean porosity with a random perturbation, which can be either uniformly^43^ or normally^61^ distributed. As a result, the porosity field generated by this method is mesh dependent and consequently inconsistent with a real rock porosity distribution, especially for a 3-D radial mesh because its size typically increases with increasing radius. Ratnakar *et al*.^50^ proposed a method to generate same porosity field for different cases when they performed simulation on 3-D linear cores. Here, similar technique is used to generate the initial porosity field in radial domain. Specifically, some discrete points, which are distributed uniformly in the physical domain, are generated first, and the porosity values is assigned to these points by adding a random perturbation to the average value of porosity *ϕ*_0_. Similar to previous works, the random perturbation is assumed to be uniformly distributed in the interval $[-{\rm{\Delta }}{\varphi }_{0},{\rm{\Delta }}{\varphi }_{0}]$. Then, the whole porosity field related to the computational grids is obtained by interpolation based on these discrete points and their values. Obviously, the density of the initial discrete points determines the correlation length, and the heterogeneity magnitude is the same as the magnitude of the perturbation. Moreover, because the distance between the initial discrete points characterizes the correlation length of the porosity field, an anisotropic porosity field can be generated by varying the density of the basal points in *x*, *y* and *z* directions. Compared with previous works, the method described above makes the generated porosity field more realistic in space and independent of the grid size.

The correlations used to calculate the mass transfer coefficient and the effective dispersion coefficients are adopted here as follows^44^:

11 $$Sh=\frac{2{k}_{c}{r}_{p}}{{D}_{m}}=S{h}_{\infty }+0.7{\mathrm{Re}}_{p}^{1/2}S{c}^{1/3}$$

12 $${D}_{eR}={\alpha }_{os}{D}_{m}+\frac{2{\lambda }_{R}|{\bf{u}}|{r}_{p}}{\varphi }$$

13 $${D}_{eT}={\alpha }_{os}{D}_{m}+\frac{2{\lambda }_{T}|{\bf{u}}|{r}_{p}}{\varphi }$$

where *Sh* is the Sherwood number and represents dimensionless mass transfer coefficient; *sh*_∞_ is the asymptotic Sherwood number; *D*_*m*_ is the effective molecular diffusivity of acid; *Re*_*p*_ is the pore Reynolds number defined as ${\mathrm{Re}}_{p}=2u{r}_{p}/\nu$, *v* is the kinematic viscosity; *Sc* is the Schmidt number defined as $Sc=\nu /{D}_{m}$; and *D*_*eR*_ is the longitudinal dispersion coefficient in *r* direction; *D*_*eT*_ is the transverse dispersion coefficient in *θ* and *z* direction; |**u**| is the magnitude of fluid velocity; *α*_*os*_, *λ*_*R*_ and *λ*_*T*_ are constants that depend upon the pore structure, and have typical values of 0.5, 0.5, 0.1 for a packed-bed of spheres, respectively^43,67^.

To complete the Darcy scale model, some appropriate structure-property relations, named the core scale model, are required to capture the evolution in permeability, pore radius, and specific area with changing porosity. As mentioned in the preceding section, the relations used in previous studies are not general. Here, the permeability of the medium is related to its local porosity using the analytical expression obtained by Xu and Yu^63^ based on the fractal geometry theory. It is given by

14 $$K=\frac{{(\pi {D}_{f})}^{\frac{(1-{D}_{T})}{2}}{(8-4{D}_{f})}^{\frac{(1+{D}_{T})}{2}}{d}^{2}}{128(3+{D}_{T}-{D}_{f})}{(\frac{\varphi }{1-\varphi })}^{\frac{3+{D}_{T}}{2}}$$

where *D*_*f*_ and *D*_*T*_ are fractal dimension for pore spaces and tortuosity, respectively. According to Xu and Yu^63^, they can be determined by

15 $${D}_{f}={d}_{E}-\frac{\mathrm{ln}\,\varphi }{\mathrm{ln}\,\frac{{\lambda }_{\min }}{{\lambda }_{\max }}}$$

16 $${D}_{T}=1+\frac{\mathrm{ln}(1-\frac{\varphi }{2}+\frac{\sqrt{1-\varphi }}{4}+\frac{(\varphi +1+\sqrt{1-\varphi })\cdot \sqrt{9-5\varphi -8\sqrt{1-\varphi }}}{8\varphi })}{\mathrm{ln}(\frac{{D}_{f}-1}{\sqrt{{D}_{f}}}\frac{{\lambda }_{\max }}{{\lambda }_{\min }}\sqrt{\frac{1-\varphi }{\varphi }\frac{\pi }{8-4{D}_{f}}})}$$

where *d*_*E*_ is the Euclidean dimension, and they have values of 2 in the two-dimensional space and 3 in the three-dimensional space; *λ*_min_ and *λ*_max_ are the smallest pore diameter and the largest diameter, respectively. The value of *λ*_min_/*λ*_max_ can be calculated by an analytic expression with porosity as the variable^70^. Xu and Yu^63^ presented a sensitivity analysis with respect to the *λ*_min_/*λ*_max_, and the results show that the value of *λ*_min_/*λ*_max_ has little influence on the fractal dimension (see Supplementary Fig. S2). Therefore, for simplicity, the value of *λ*_min_/*λ*_max_ is taken as 0.01 in this paper, which is selected from Xu and Yu’s work^63^.

Equation (14) presents a relation between the porosity and permeability, and no empirical constant is involved. In addition, Xu and Yu^63^ have verified that the results calculated from Equation (14) are consistent with those by Happel and Brenner^64^, Eidsath, *et al*.^71^, Rahli, *et al*.^72^, Davies and Dollimore^73^, Kyan, *et al*.^74^. The permeability, average pore radius, and specific surface area are related to their initial values *K*_0_, *r*_0_, and *a*_0_, respectively by

17 $$\frac{K}{{K}_{0}}=\frac{{(\pi {D}_{f})}^{\frac{(1-{D}_{T})}{2}}{(8-4{D}_{f})}^{\frac{(1+{D}_{T})}{2}}(3+{D}_{T0}-{D}_{f0})}{{(\pi {D}_{f0})}^{\frac{(1-{D}_{T0})}{2}}{(8-4{D}_{f0})}^{\frac{(1+{D}_{T0})}{2}}(3+{D}_{T}-{D}_{f})}{(\frac{\varphi }{1-\varphi })}^{\frac{3+{D}_{T}}{2}}{(\frac{{\varphi }_{0}}{1-{\varphi }_{0}})}^{-\frac{3+{D}_{T0}}{2}}$$

18 $$\frac{{r}_{p}}{{r}_{0}}=\sqrt{\frac{K{\varphi }_{0}}{{K}_{0}\varphi }}$$

19 $$\frac{{a}_{v}}{{a}_{0}}=\frac{\varphi {r}_{0}}{{\varphi }_{0}{r}_{p}}$$

Because the rock is heterogeneous and the constant-injection rate condition is used at the inlet boundary, the flux should be assigned according to the permeability of the grid at the inlet boundary when numerical simulation is performed. However, in previous studies, a constant injection velocity condition is imposed at the inlet boundary, which means that all boundary grids have the same injection velocity, although the permeability of each grid cell is different. As a result, the convergence rate at the early stage of the simulation is poor. To improve the efficiency, the medium in question is extended by adding a homogeneous porous medium, which has a thickness of Δ*r* and porosity of 0.99, to its injection end (see Supplementary Fig. S1). After this extension, the injected acid flows into the extended medium at constant velocity and then diverts into the primary model domain according to its local permeability.

We discretize the governing equations using the finite volume method in a 3D radial grid system. The diffusion term is discretized using the central difference scheme and the convection term is discretized using the upwind scheme, which guarantees the stability of the numerical formulation. The grid size is determined on the basis of *PV*_*BT*_. The grid size is refined until the *PV*_*BT*_ becomes insensitive to any grid changes. The pressure and velocity field are obtained first by solving the continuity equation. And then, the concentration and porosity field are updated by solving the mass balance equation and the reaction equation, using the operator splitting method combined with an extrapolation technique, as discussed in Maheshwari, *et al*.^33^.

### Data availability

The datasets generated during the current study are available from the corresponding author on reasonable request.

## Conclusions

The main contribution of this work is the modelling and simulation of reactive dissolution patterns in the 3D radial flow condition. Specifically, a new structure-property relationship is developed to complete the Darcy scale model. The simulation result from the present model is in good agreement with the available experimental results. By analysing the effect of injection rate, heterogeneity magnitude, and correlation length, on the dissolution process, the following conclusions are made:

1. The dissolution patterns (face dissolution, conical wormhole, wormhole, ramified wormhole, and uniform dissolution) observed in experiments are obtained from simulation under the 3-D radial flow condition.
2. With the increase of heterogeneity magnitude, wormholes become highly branched and the number of dominate wormholes decreases. At appropriate heterogeneity magnitude, only one dominate wormhole forms.
3. The correlation length has no effect on the number of dominate wormholes, but influences the wormhole diameter and its branchi-ness.

It should be noted that the model presented in this work is completely general, although we only focus on the dissolution of carbonate rock treated with HCl. Therefore, the model can be extended to study other solvent-mineral systems by changing the reaction kinetics. One potential application is to investigate the CO_2_ induced dissolution in CO_2_ sequestration, in which the Darcy’s flow should be extended to describe the multiphase flow, and another concertation mode may be needed to describe the nonlinear kinetics. The model can also be extended to study the precipitation problem by changing the source term. Some of these extensions will be pursued in future work.

## Electronic supplementary material

Supplementary material
