# Supplementary material for: 3-D Modelling and Experimental Comparison of Reactive Flow in Carbonates under Radial Flow Conditions
Source: Sci Rep. 2017 Dec 18;7:17711. doi: 10.1038/s41598-017-18095-2 (PMC5735102; doi:10.1038/s41598-017-18095-2)
Supplement: Supplementary file 1 — Supplementary material [file 41598_2017_18095_MOESM1_ESM.pdf]

Supplementary Information:
3-D Modelling and Experimental Comparison of Reactive Flow in Carbonates under Radial Flow Conditions

Piyang Liu[a,b], Jun Yao[a]*, Gary Douglas Couples[b], Jingsheng Ma[b], Oleg Iliev[c]

[a] School of Petroleum Engineering, China University of Petroleum (East China), QingDao, China

[b] Institute of Petroleum Engineering, Heriot-Watt University, Riccarton, Edinburgh, UK

[c] Fraunhofer Institute for Industrial Mathematics (ITWM), Kaiserslautern, 67663, Germany

*Corresponding Email:rcogfr_upc@126.com

Table of contents

Dimensionless models S2

Numerical scheme.. S3

Extension of the heterogeneous porous medium in question.................................... S5

Variation of permeability with porosity for different values of $\lambda_{\min}/\lambda_{\max}$ S6

Breakthrough curve for various dissolution patterns S7

Reference S7

List of figures

Fig. S1 Schematic of the extension of the heterogeneous porous medium in question. S6

Fig. S2 Effect of $\lambda_{\min}/\lambda_{\max}$ on the permeability variation with porosity changing...... S6

Fig. S3 Breakthrough volume in Figure 2 are plotted as function of the reciprocal of Damköhler number S7

List of tables

Table S1 List of parameters and dimensionless numbers used in simulation............ S5

Dimensionless models

Simulations are performed on dimensionless models. To non-dimensionalize the mathematical model, the following dimensionless variables and parameters are defined

$$r_D = \frac{r}{r_e}\ ,\quad H_D = \frac{H}{r_e}\ ,\quad r_{wD} = \frac{r_w}{r_e}\ ,\quad \mathbf{U} = \frac{\mathbf{u}}{u_0}\ ,\quad t_D = \frac{t}{(r_e/u_0)}\ ,\quad \boldsymbol{\kappa} = \frac{\mathbf{K}}{K_0}\ ,\quad r_{pD} = \frac{r_p}{r_{p0}}\ ,\quad a_{vD} = \frac{a_v}{a_0}\ ,$$

$$\mathbf{D} = \frac{\mathbf{D}_e}{D_m}\ ,\quad C_{fD} = \frac{C_f}{C_0}\ ,\quad C_{sD} = \frac{C_s}{C_0}\ ,\quad P_D = \frac{P - P_e}{(\mu u_0 r_e)/K_0}\ ,\quad Th^2 = \frac{2R_s r_{p0}}{D_{m0}}\ ,\quad Da = \frac{R_s a_0 r_e}{u_0}\ ,$$

$$N_{ac} = \frac{\alpha_c C_0}{\rho_s}\ ,\quad Pe_L = \frac{u_0 r_e}{D_{m0}}\ ,\quad \eta = \frac{2r_{p0}}{r_e}\ ,\quad \Phi^2 = \frac{R_s a_0 r_e^2}{D_m}\ .$$

where the subscript D represents dimensionless variable; r_e is the external radius; H is the height of the domain; r_w is the internal radius; **U** is dimensionless velocities vector; u_0 is the inlet velocity; **κ** is the dimensionless permeability tensor; **D** is the dimensionless dispersion tensor; C_0 is the inlet concentration of the acid; P_e is the pressure at the exit boundary; η is the pore-to-domain scale ratio; The parameters obtained from the non-dimensionalization are the pore scale Thiele modulus h_T^2, defined as the ratio of diffusion time to reaction time, Damköhler number Da, defined as the ratio of convection time to reaction time, the axial Peclet number Pe_L, defined as the ratio of advective transport rate to diffusive transport rate, the acid capacity number N_{ac}, defined as the volume of solid dissolved per unit volume of acid consumed, and the macroscopic Thiele modulus Φ^2 is the core-scale equivalent of the pore-scale Thiele modulus. More details on the definition of these dimensionless groups can be found in Panga, et al. [1] and Kalia and Balakotaiah [2]. The partial differential equations (PDEs), after conversion into the non-dimensionalized form, are as follows:

$$(U_r, U_\theta, U_z) = -\kappa\left(\frac{\partial P_D}{\partial r_D}, \frac{1}{r_D}\frac{\partial P_D}{\partial \theta}, \frac{\partial P_D}{\partial z_D}\right) \tag{S1}$$

$$\nabla\cdot\left(\kappa\frac{\partial P_D}{\partial r_D}+\kappa\frac{1}{r_D}\frac{\partial P_D}{\partial\theta}+\kappa\frac{\partial P_D}{\partial z_D}\right)=\frac{N_{ac}Da\cdot a_{vD}C_{fD}}{\left(1+\frac{Th^2 r_{pD}}{Sh}\right)} \tag{S2}$$

$$\begin{aligned}&\frac{\partial}{\partial t_D}\left(\phi C_{fD}\right)+\frac{1}{r_D}\frac{\partial}{\partial r_D}\left(r_D U_r C_{fD}\right)+\frac{1}{r_D}\frac{\partial}{\partial\theta}\left(U_\theta C_{fD}\right)+\frac{\partial}{\partial z_D}\left(U_z C_{fD}\right)\\&=\frac{1}{r_D}\frac{\partial}{\partial r_D}\left[r_D\left(\frac{\phi\alpha_{os}Da}{\Phi^2}+\lambda_R|\mathbf{U}|r_{pD}\eta\right)\frac{\partial C_{fD}}{\partial r_D}\right]+\frac{1}{r_D^2}\frac{\partial}{\partial\theta}\left[\left(\frac{\phi\alpha_{os}Da}{\Phi^2}+\lambda_T|\mathbf{U}|r_{pD}\eta\right)\frac{\partial C_{fD}}{\partial\theta}\right]\\&+\frac{\partial}{\partial z_D}\left[\left(\frac{\phi\alpha_{os}Da}{\Phi^2}+\lambda_T|\mathbf{U}|r_{pD}\eta\right)\frac{\partial C_{fD}}{\partial z_D}\right]-\frac{Da\cdot a_{vD}C_{fD}}{\left(1+\frac{Th^2 r_{pD}}{Sh}\right)}\end{aligned} \tag{S3}$$

$$\frac{\partial\phi}{\partial t_D}=\frac{N_{ac}Da\cdot a_{vD}C_{fD}}{\left(1+\frac{Th^2 r_{pD}}{Sh}\right)} \tag{S4}$$

And the resulting boundary and initial conditions are

$$\begin{cases}-\kappa\left.\frac{\partial P_D}{\partial r_D}\right|_{r_D=r_{wD}}=1,\ P_D\big|_{r_D=1}=0,\ P_D\left(r_D,0,z_D\right)=P_D\left(r_D,2\pi,z_D\right),\\-\kappa\left.\frac{\partial P_D}{\partial z_D}\right|_{z_D=0,H}=0,\ \left.\frac{\partial C_{fD}}{\partial z_D}\right|_{z_D=0,H}=0,C_{fD}\left(r_D,0,z_D\right)=C_{fD}\left(r_D,2\pi,z_D\right),\\C_{fD}\big|_{r_D=r_{wD}}-\left(\frac{\phi\alpha_{os}Da}{\Phi^2}+\lambda_R|\mathbf{U}|r_{pD}\eta\right)\left.\frac{\partial C_{fD}}{\partial r_D}\right|_{D=r_{wD}}=1,\ \left.\frac{\partial C_{fD}}{\partial r_D}\right|_{r_D=1}=0.\end{cases} \tag{S5}$$

Numerical scheme

The finite volume method is used in this work to discretize the reactive flow equations which describe the fluid flow, solute transport and rock dissolution in porous media. To get a stable numerical scheme, the diffusion term and convection term are discretized using the second order central difference scheme and the upwind scheme, respectively. For the transient term, we use the Backward Euler scheme to discretize. Therefore the accuracy of the numerical scheme in time and space is first order. To obtain the solution efficiently, we use the operator splitting method combined with extrapolation technology as discussed in Maheshwari, et al. [3]. As Maheshwari [5] mentioned, by using the extrapolation technology, the accuracy of the numerical scheme in time becomes second order. Firstly, the pressure field is obtained by solving Eq. (S2) using the initial concentration and porosity fields. And then, the

velocity field is determined by Eq. (S1). Using the updated velocity field, the mass transfer coefficient and the effective dispersion coefficients are updated, and substituted into Eq. (S3). Finally, Eq. (S3) and Eq. (S4) are solved using the operator splitting method. For convenience, we rewrite Eq. (S3) and Eq. (S4) as

$$\frac{\partial \mathbf{F}}{\partial t} = \mathbf{L}_{CD}\mathbf{F} + \mathbf{L}_{R}\mathbf{F} \tag{S6}$$

where, $\mathbf{F} = \left[\phi C_{fD} + \phi / N_{ac}, \phi\right]^T$. Performing the diffusion-convection operator $\mathbf{L}_{CD}$ and reaction operator $\mathbf{L}_R$ to $\mathbf{F}$, we get

$$\mathbf{L}_{CD}\mathbf{F} = \begin{bmatrix} \left(\begin{array}{l} \frac{1}{r_D}\frac{\partial}{\partial r_D}\left(r_D U_r C_{fD}\right) + \frac{1}{r_D}\frac{\partial}{\partial \theta}\left(U_\theta C_{fD}\right) + \frac{\partial}{\partial z_D}\left(U_z C_{fD}\right) \\ -\frac{1}{r_D}\frac{\partial}{\partial r_D}\left[r_D \left(\frac{\phi \alpha_{os} Da}{\Phi^2} + \lambda_R \left|\mathbf{U}\right| r_{pD}\eta \right)\frac{\partial C_{fD}}{\partial r_D} \right] \\ -\frac{1}{r_D^2}\frac{\partial}{\partial \theta}\left[\left(\frac{\phi \alpha_{os} Da}{\Phi^2} + \lambda_T \left|\mathbf{U}\right| r_{pD}\eta \right)\frac{\partial C_{fD}}{\partial \theta} \right] \\ -\frac{\partial}{\partial z_D}\left[\left(\frac{\phi \alpha_{os} Da}{\Phi^2} + \lambda_T \left|\mathbf{U}\right| r_{pD}\eta \right)\frac{\partial C_{fD}}{\partial z_D} \right] \end{array} \right) \\ 0 \end{bmatrix} \tag{S7}$$

$$\mathbf{L}_{R}\mathbf{F} = \begin{bmatrix} 0 \\ N_{ac} Da \cdot a_{vD} \left(1 + \frac{Th^2 r_{pD}}{Sh}\right)^{-1} C_{fD} \end{bmatrix} \tag{S8}$$

Using the operator splitting method, the diffusion-convection operator is solved first

$$\frac{\mathbf{F}^{n+1/2} - \mathbf{F}^{n}}{\Delta t} = \left\lfloor \mathbf{L}_{CD}\mathbf{F}^{n+1/2} \right\rfloor \tag{S9}$$

And then, the reaction operator is solved using the obtained results as

$$\frac{\mathbf{F}^{n+1} - \mathbf{F}^{n+1/2}}{\Delta t} = \left\lfloor \mathbf{L}_{R}\mathbf{F}^{n+1} \right\rfloor \tag{S10}$$

In Eq. (S9) and Eq. (S10), $\lfloor \cdot \rfloor$ represents the discretized version of the operators.

After updated the concentration and porosity field, we repeat the procedure until acid break through the rock. In our simulations, breakthrough is defined when overall permeability is increased by a factor 100. The parameters values and dimensionless numbers used in the simulations are listed in Table S1.

Table S1 List of parameters and dimensionless numbers used in simulation.

Parameter	Value
ϕ_0	0.2
$\Delta\phi$	0.15
h_T^2	0.07
Φ^2	10^6
Sh_∞	3
N_{ac}	0.1
Sc	1000
η	10^{-6}
α_{os}	0.5
λ_T	0.5
λ_R	0.1
$\lambda_{\min}/\lambda_{\max}$	0.01
K_0	2mD
r_0	1μm

Extension of the heterogeneous porous medium in question

To improve the convergence rate, the medium in question is extended by adding a homogeneous porous medium, which has a thickness of Δr and porosity of 0.99, to its injection end, as shown in Fig. S1.

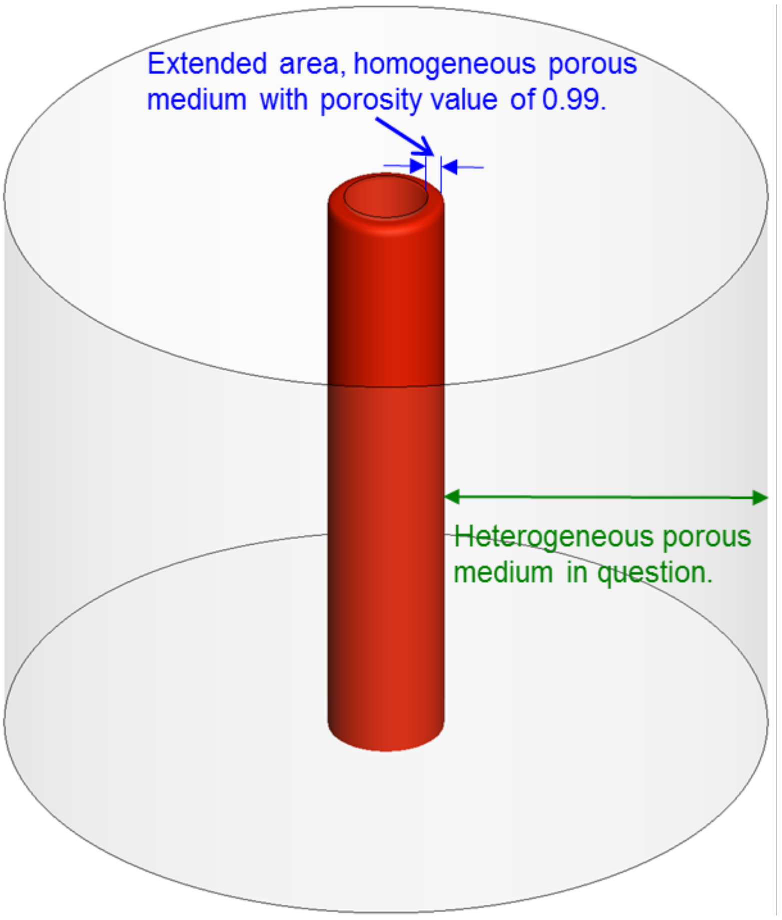

Fig. S1 Schematic of the extension of the heterogeneous porous medium in question.

Variation of permeability with porosity for different values of $\lambda_{\min}/\lambda_{\max}$

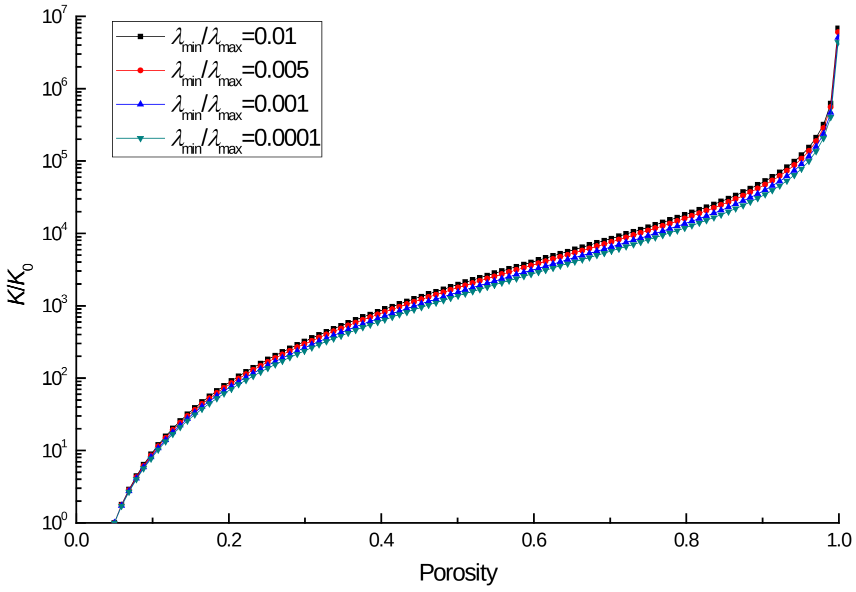

Fig. S2 Effect of $\lambda_{\min}/\lambda_{\max}$ on the permeability variation with porosity changing.

The only uncertain variable involved in our structure-property relations is $\lambda_{\min}/\lambda_{\max}$. Fig. S2 shows the effect of $\lambda_{\min}/\lambda_{\max}$ on the variation of permeability with porosity. It can be seen that the variation of permeability with porosity is insensitive to $\lambda_{\min}/\lambda_{\max}$.

Breakthrough curve for various dissolution patterns

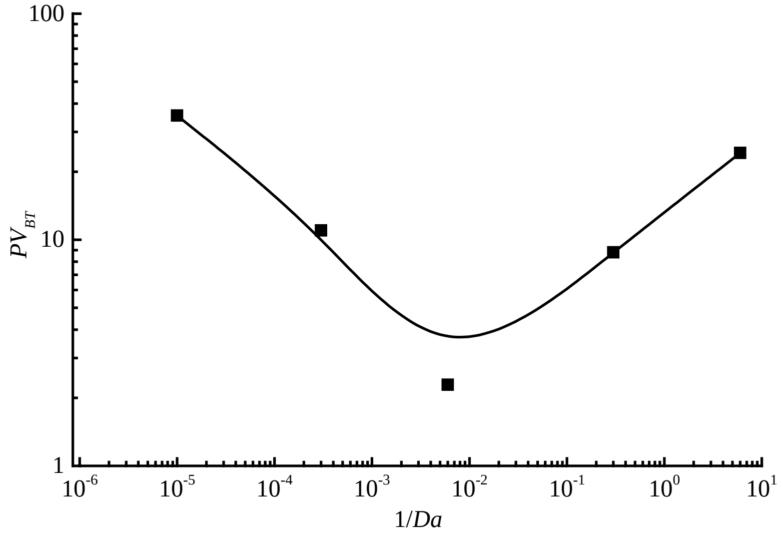

Fig. S3 Breakthrough volume in Figure 2 are plotted as function of the reciprocal of Damköhler number.

The amount of acid required to breakthrough for various dissolution patterns as shown in Figure 2 are plotted in Fig. S3 against the reciprocal of Damköhler number. It can be clearly seen that PV_{BT} is minimum when wormhole dissolution is formed, which confirms the simulation result conducted by Maheshwari et al.[3] under 3-D linear flow condition.

Reference

1 Panga, M. K., Ziauddin, M. & Balakotaiah, V. Two-scale continuum model for simulation of wormholes in carbonate acidization. *AlChE J.* **51**, 3231-3248 (2005).

2 Kalia, N. & Balakotaiah, V. Effect of medium heterogeneities on reactive dissolution of carbonates. *Chem. Eng. Sci.* **64**, 376-390 (2009).

3 Maheshwari, P., Ratnakar, R. R., Kalia, N. & Balakotaiah, V. 3-D simulation and analysis of reactive dissolution and wormhole formation in carbonate rocks. *Chem. Eng. Sci.* **90**, 258-274, doi:http://dx.doi.org/10.1016/j.ces.2012.12.032 (2013).

4 Kalia, N. & Balakotaiah, V. Modeling and analysis of wormhole formation in reactive dissolution of carbonate rocks. *Chem. Eng. Sci.* **62**, 919-928, doi:http://dx.doi.org/10.1016/j.ces.2006.10.021 (2007).

5 Maheshwari. Modeling and Simulation of Reactive Dissolution and Wormhole Formation in Carbonate Rocks. *University of Houston.* (2013)
